# AN UMBRELLA REVIEW OF SYMPTOM-SPECIFIC NONPHARMACOLOGICAL INTERVENTIONS FOR DEMENTIA

**DOI:** 10.1093/geroni/igad104.2544

**Published:** 2023-12-21

**Authors:** Eunhee Cho, Min Jung Kim, Ji Yeon Lee, Minhee Yang, Jiyoon Jang, Jungwon Cho

**Affiliations:** Yonsei University College of Nursing, Seoul, Republic of Korea; Yonsei University, Seoul, Republic of Korea; Yonsei University, Seoul, Republic of Korea; Yonsei University, Seoul, Republic of Korea; Yonsei University, Seoul, Republic of Korea; Yonsei University College of Nursing, Seoul, Republic of Korea

## Abstract

A variety of non-pharmacological interventions (NPI) have been used for managing behavioral and psychological symptoms of dementia (BPSD), but only a small effect size has been reported. Also, due to a lack of clinical guidelines, individualized BPSD interventions considering each person’s preferences and unique symptom profiles have not been available. This umbrella review aimed to summarize the current evidence of NPI for managing BPSD and to suggest an evidence-based algorithm that can guide future clinical practices on choosing an NPI targeting an individual or cluster of BPSD. We used the Cochrane methodology for umbrella reviews following a predetermined protocol registered. A total of five electronic databases (PubMed, CINAHL, Embase, PsychINFO, and Cochrane Database) was searched based on the PICO formulation. The eligibility was independently screened by two reviewers. The quality of the individual review was evaluated by the two reviewers using the A Measurement Tool to Assess Systematic Reviews 2 (AMSTAR2). A total of 32 reviews were included. The reviews collectively indicated that NPI have shown mixed results in managing an individual or cluster of BPSD. As to the NPI, a wide variety was observed in which a music therapy was most commonly used. Of the various BPSD symptoms, affective/hyperactivity symptoms such as depression, anxiety, and agitation have been most investigated. Based on the effect sizes and quality appraisal results, we created an evidence-based algorithm that can provide several NPI options for each sub-category of BPSD. Future studies are required to provide a more comprehensive clinical practice algorithm.

